# Assessing Medical Student Perceptions of Open-Book Exams for Self-Directed Learning

**DOI:** 10.7759/cureus.59218

**Published:** 2024-04-28

**Authors:** Ali G Alghamdi

**Affiliations:** 1 College of Medicine, Al Baha University, Al Baha, SAU

**Keywords:** student performance analysis, assessment fairness, assessment strategies strategies, stress reduction, exam preparation methods, self-directed learning, open-book exams

## Abstract

Introduction

The landscape of medical education is constantly evolving, with innovative assessment methods being integrated to better align with the requirements of modern healthcare education. Among these, open-book exams (OBEs) represent a significant shift from traditional closed-book exams (CBEs), promising to enhance learning outcomes and better evaluate students' understanding of medical concepts. This study aims to explore the multifaceted impact of OBEs on medical students, including their perceptions, study behaviors, stress levels, and the cultivation of critical thinking and self-directed learning skills.

Methodology

This is a cross-sectional study, which utilized a mixed-methods approach, conducted at Al Baha University's College of Medicine, to explore the impact of OBEs on self-directed learning among 129 medical students over a 15-day period in October 2023. The research combined quantitative data from online questionnaires, assessing students' experiences, stress, understanding, and study strategies, with qualitative insights from in-depth interviews and open-ended survey questions. Participants were final-year medical students with prior experience in OBEs, selected to minimize bias. Data analysis was performed using Statistical Product and Service Solutions (SPSS, version 25; IBM SPSS Statistics for Windows, Armonk), focusing on descriptive and inferential statistics, while qualitative data underwent thematic analysis to identify patterns in students' perceptions of self-directed learning opportunities. The study was ethically approved, ensuring participant confidentiality and informed consent.

Result

Regarding the medical student perspectives on OBEs, the study revealed that the majority of medical students strongly perceive OBEs as less stressful (77, 59.69%) and easier to prepare for (79, 61.24%) compared to traditional exams. A significant proportion also believe OBEs accurately assess their comprehension (106, 82.17%) and prefer them as a mode of assessment (106, 82.17%). Furthermore, most students (87, 67.44%) reported performing better on OBEs compared to CBEs. Regarding the assessment of self-directed learning using the OBE method, students predominantly utilized highlighting important points (70, 54.26%) as a preparation method for OBEs. A large majority (85, 65.89%) considered OBEs as a fair assessment of self-directed learning and believed that they encourage self-directed learning (114, 88.37%).

Conclusion

OBEs represent a promising direction for medical education, offering a way to better prepare students for the complexities of real-world medical practice. Future strategies should include not only the refinement of OBE methodologies but also the integration of practice opportunities that enable students to hone their skills in applying knowledge effectively.

## Introduction

In the field of medical education, various methods of assessing students' knowledge and understanding have been used. Examples include classroom assessment techniques, essay questions, patient management problems, modified essay questions (MEQs), checklists, objective structured clinical examination (OSCE), etc. One of these methods is the open-book exam (OBE), which allows students to refer to their notes, textbooks, or other materials during the examination.

In our institution's curriculum, we have implemented various types of teaching methods, such as problem-based learning, team-based learning, and self-directed learning. Self-directed learning is one of these methods, wherein students take the initiative to diagnose their learning needs, set learning goals, identify resources, search for learning strategies, and evaluate outcomes independently or with minimal guidance from others [1].

Numerous studies have delved into the use of OBEs in the field of medical education [2-6]. For instance, Agarwal et al. conducted a study comparing the effects of open- and closed-book tests on learning, revealing that OBEs might facilitate better long-term retention of information than closed-book exams (CBEs) [7]. This implies that open-book assessments could serve as a valuable method for evaluating students' grasp of medical concepts.

Research has shown that students preparing for CBEs tend to focus on memorization and assigned texts [2]. However, a novel approach, OBEs, has emerged, providing students with access to their course materials, textbooks, and notes during the evaluation process [8]. This shift aligns with the evolving demands of modern healthcare education, which prioritize self-directed learning skills among medical students [9].

Several studies have investigated the study behavior and outcomes associated with both CBEs and OBEs [2,7,9,10]. One study determined that CBEs necessitate students to rely on their memory and recall, while OBEs permit the use of available resources to answer test questions [2]. Another study explored the impact of open- and closed-book tests on medical students' learning approaches and indicated that closed-book tests may encourage deep learning and enhance students' confidence in their preparation, whereas open-book tests may motivate students to engage in more extensive study efforts [3].

OBEs hold promise for enhancing students' learning and self-efficacy. They stimulate higher-order cognitive processes, such as evaluation and creation, in contrast to traditional CBEs that center on lower-order cognitive functions such as reproduction and description [9]. Furthermore, OBEs have the potential to foster the cultivation of effective study strategies such as critical thinking, information synthesis, and problem-solving skills, as well as enhance students' self-directed learning.

The study's main objective was to investigate various topics related to students' experiences taking OBEs. By investigating student experiences, stress levels, comprehension of the subject matter, study strategies used, influence on self-directed learning, and preferences in the context of OBEs, the study aimed to obtain a thorough understanding. The study examined how students felt about OBEs and examined their experiences with them. It sought to elucidate the advantages and difficulties related to this evaluation technique. The experiment also assessed stress levels by contrasting open-book tests with conventional CBEs. The study aimed to pinpoint the causes of stress in both types of assessments. An important component was determining how well OBEs assessed students' knowledge and comprehension of the subject. This test aimed to show how well this assessment method measures students' understanding. In addition, the study examined how students studied for open-book tests vs the typical CBEs to identify any variations in methodology. It also aimed to investigate the effects of OBEs on independent learning and critical thinking abilities, which are essential components of medical practice.

Moreover, this study aimed to comprehend the students' viewpoints concerning the fairness of OBEs in assessing self-directed learning. It also examined how students thought they performed in open-book tests instead of CBEs, providing more information about their comfort level and aptitude for this type of evaluation.

## Materials and methods

This research was undertaken among the medical students of Al Baha University, Saudi Arabia, over a 15-day period, conducted electronically from October 01 to October 15, 2023. It encompassed 129 medical students at Al Baha University.

This study employed a mixed-methods approach to explore the impact of OBEs on self-directed learning among medical students. The methodology was divided into two parts: a quantitative assessment via an online questionnaire covering student experiences, stress, understanding, study strategies, and preferences; and a qualitative analysis based on open-ended questions and in-depth structured interviews with students who had taken OBEs.

This comprehensive approach, blending qualitative and quantitative analyses, provided a detailed view of students' perspectives on OBEs [11].

Medical student experience with OBEs (Section 1)

The study engaged 129 medical students from Al Baha University as participants to gauge their experiences with OBEs. Employing an online questionnaire (see Appendix), the data collection method incorporated various queries comprising multiple-choice and Likert scale questions. This questionnaire was structured to extract multifaceted insights into the students' encounters with OBEs, encompassing their perceptions, stress levels, study techniques, and preferences.

A quantitative technique was used to examine the collected data, utilizing descriptive and inferential statistics. The questionnaire responses were processed and analyzed using statistical approaches to identify patterns, trends, and relationships within the dataset. The fundamental characteristics of the data, such as mean responses, frequency distributions, and core patterns, were summarized by descriptive statistics. Based on the sample gathered, inferential statistics were used to draw conclusions and forecasts about the larger student population. This thorough methodology provided a statistical basis to identify trends, variances, and correlations in the participants' views and attitudes regarding this assessment method, enabling a detailed comprehension of the quantitative components of their experiences with OBEs.

Assessment of self-directed learning using the OBE method (Section 2)

A focused analysis of the characteristics of self-directed learning associated with OBEs was carried out. This portion involved 129 medical students with firsthand experience with OBEs. The approach was focused on gathering quantitative and some qualitative data in order to gain a better understanding of the students' self-directed learning experiences during OBEs. In-depth interviews and additional open-ended questions incorporated into the online survey were used in the data collection procedure (see Appendix). These two methods offered a comprehensive and multifaceted investigation of the participants' beliefs, dispositions, and actions concerning independent learning within the framework of open-book tests.

Study area: This research was carried out at Al Baha University, College of Medicine.

Inclusion and exclusion criteria: Inclusion criteria for this study comprised medical students enrolled at Al Baha University from fifth and sixth years who consented to take part and had experienced OBEs as a mode of assessment for self-directed learning. Exclusion criteria included individuals who declined participation and those who had not undergone it.

Sample size and sampling technique: A total of 165 medical students were invited to participate. Only 129 students were accepted to participate in this survey. The sample size was determined using the Raosoft sample size calculator (http://www.raosoft.com), using 5% as a margin of error, 95% as a confidence interval, and 50% as response distribution. The calculated sample size was 116.

Data collection: Participants were asked to complete a questionnaire (Appendix 1) sent to them after completing the exam through WhatsApp. To mitigate bias, only medical students in their final years were selected. Additionally, a brief lecture was provided outlining the purpose of the survey and how to accurately respond to the questions. Emphasis was placed on selecting the most appropriate answers to ensure the integrity of the data collected. In the data collection process, both in-depth interviews and additional open-ended questions included in the online survey were utilized. The response rate was 78.18%.

Data entry and analysis: The data collection process commenced with the utilization of Google Forms. Subsequent to this initial collection, the data were downloaded in the form of an Excel spreadsheet. For the purpose of in-depth analysis, this spreadsheet was then exported into specialized analytical software. Statistical Product and Service Solutions (SPSS, version 25; IBM SPSS Statistics for Windows, Armonk, NY) was used to analyze the data. Our primary step in the analytical phase involved conducting a descriptive analysis, where data were presented in terms of frequencies and percentages. To bolster credibility, measures such as the mean, mode, and standard deviation were computed. These statistical analyses provide insight into the central tendency, frequency, and dispersion of the data, respectively, thus strengthening the robustness of the findings.

Thematic analysis was used for the questionnaire's open-ended answers and the data gathered from the interviews. This approach made it possible to find recurrent themes, patterns, and underlying ideas in the qualitative data. This resulted in thoroughly comprehending the complex viewpoints and self-directed learning opportunities associated with OBEs.

A Likert scale was employed to evaluate the majority of independent variables. Each category was assigned a numerical value for analytical purposes as follows: "5" for Strongly agree, "4" for Agree, "3" for Neutral, "2" for Disagree, and "1" for Strongly disagree. This adjustment ensures consistency with statistical conventions, facilitating the calculation of means and standard deviations.

Reliability analysis: The value associated with Cronbach's alpha for the variables is 0.89. Cronbach's alpha is a measure of the internal consistency or reliability of a set of items or questions in a survey or test. In this case, the variable has Cronbach's alpha value of 0.89, indicating a relatively high level of internal consistency among the items related to the applicable variable.

Ethical considerations

Participants provided their consent after being informed of the study's objectives and aims on the initial page of the questionnaire. To ensure confidentiality, any personal data that could reveal their identities were excluded from the questionnaires. The study obtained ethical approval from the Research Ethics Committee (REC) at the College of Medicine, Al Baha University, Al Baha, Saudi Arabia, with approval no.: REC/SUR/BU-FM/2023/83. Throughout the study, all participants' anonymity was strictly preserved.

## Results

Medical students' experience with OBEs

The primary conclusions derived from Section 1 of the study yield crucial insights into the experiences and perspectives of medical students concerning OBEs. Firstly, a noteworthy finding pertains to participants' stress levels: Strongly Agree: 77 (59.69%), Agree: 29 (22.48%), Neutral: 10 (7.75%), Disagree: 5 (3.88%), and Strongly Disagree: 8 (6.2%). The data indicate a prevalent sentiment among students, with 77 (59.69%) strongly asserting that OBEs induce less stress than traditional exams. OBEs had a mean stress level of 4.26, a mode of 5, and a standard deviation of 1.15. This suggests they are perceived as less stressful compared to traditional exams This aligns with the idea that access to reference materials can alleviate anxiety associated with assessments, suggesting a positive reception of the open-book format.

The collected data reflect participants' perceptions regarding the effectiveness of OBEs in accurately assessing their knowledge and understanding. The majority of participants expressed a favorable view: 71 (55.04%) 'Strongly Agree' and 35 (27.13%) 'Agree' with the statement, collectively indicating that 82.17% support the efficacy of OBEs. A smaller proportion of participants remains neutral, with 16 respondents (12.4%) neither agreeing nor disagreeing. Opposition to the statement is minimal, with four (3.1%) disagreeing and an additional three (2.33%) strongly disagreeing. This distribution underscores a strong agreement supporting OBEs as a valid approach for assessing students' understanding of the material.

Concerning the ease of preparation, a significant number (79, 61.24%) of students strongly agree that OBEs are easier to prepare for compared to CBEs, with an additional 31 (24.03%) agreeing with this sentiment. In contrast, only a minimal number (8, 6.2%) express disagreement or strong disagreement with the perception that OBEs offer a more straightforward preparation process. The finding indicates that a significant majority of students believe OBEs are easier to prepare for than CBEs, with minimal disagreement.

In evaluating the preference for OBEs over traditional exams, the majority of students, comprising 68 (52.71%) strongly agree that OBEs provide a more effective platform to demonstrate their knowledge. An additional 25 (19.38%) express agreement with this perspective. Conversely, a relatively small percentage (9, 6.98%), either disagrees or strongly disagrees with the notion that OBEs allow for a better demonstration of their knowledge compared to traditional exams. These findings suggest a notable inclination among students towards OBEs as a preferred mode for showcasing their understanding and mastery of the subject matter.

The distribution of responses on OBEs' fairness in assessing student knowledge indicates a positive trend. A significant portion of respondents, with 57 (44.19%) strongly agreeing and 28 (21.71%) agreeing, supports the fairness of OBEs. Neutral responses stand at 25 (19.38%), 13 (10.08%) disagree, and six (4.56%) strongly disagree. The statistical summary, featuring a mean of 3.91 and a mode of 5, coupled with a standard deviation of 1.21, underscores this trend towards agreement. Despite some response variability, the predominant sentiment among participants is that OBEs are a fair method for evaluating student knowledge, with a majority expressing approval of this assessment format.

Regarding OBEs promoting critical thinking, 80 (62.02%) strongly agree, 22 (17.05%) agree, 22 (17.05%) are neutral, three (2.33%) disagree, and two (1.55%) strongly disagree. These data suggest that a substantial majority of respondents view OBEs as effective in promoting critical thinking, with over 79.07% either agreeing or strongly agreeing. The small percentage of neutrality and disagreement indicates minor reservations about this viewpoint. Overall, the findings point to a strong belief in the positive impact of OBEs on critical thinking skills (Table 1).

**Table 1:** Perspectives of students regarding open-book examinations

Student perspectives	Strongly agree (%)	Agree (%)	Neutral (%)	Disagree (%)	Strongly disagree (%)	Total (%)	Mean	Mode	Std. Deviation	*Cronbach's alpha
Open-book exams are less stressful than traditional exams.	77 (59.69%)	29 (22.48%)	10 (7.75%)	5 (3.88%)	8 (6.2%)	129 (100%)	4.26	5	1.15	0.87 (number of items: 6)
Open-book exams accurately assess my knowledge and understanding of the material.	71 (55.04%)	35 (27.13%)	16 (12.4%)	4 (3.1%)	3 (2.33%)	129 (100%)	4.29	5	0.96	
It is easier to prepare for an open-book exam compared to a closed-book exam.	79 (61.24%)	31 (24.03%)	11 (8.53%)	4 (3.1%)	4 (3.1%)	129 (100%)	4.37	5	0.98	
Open-book exams allow you to demonstrate your knowledge better than traditional exams.	68 (52.71%)	25 (19.38%)	27 (20.93%)	5 (3.88%)	4 (3.1%)	129 (100%)	4.15	5	1.08	
Open-book exams are a fair way to assess student knowledge.	57 (44.19%)	28 (21.71%)	25 (19.38%)	13 (10.08%)	6 (4.65%)	129 (100%)	3.91	5	1.21	
The open-book format encourages you to think more critically about the material.	80 (62.02%)	22 (17.05%)	22 (17.05%)	3 (2.33%)	2 (1.55%)	129 (100%)	4.36	5	0.95	

Additionally, 92 (71.32%) of respondents reported studying less for OBEs than for CBEs, while 24 (18.6%) claimed to spend the same amount of time studying, with a mean study time ratio of 1.39 and a standard deviation of 0.67. These findings suggest a prevalent perception among participants that studying for OBEs requires less time investment than for traditional CBEs (Table 2).

**Table 2:** Time spent for studying in both methods

How much time did you spend studying for the open-book exam compared to a traditional closed-book exam?	N	%	Mean	Std. deviation
I spend less time studying for the open-book exam compared to a traditional closed-book exam.	92	71.32%	1.39	0.67
I spend the same time studying for the open-book exam compared to a traditional closed-book exam.	24	18.6%		
I spend more time studying for the open-book exam compared to a traditional closed-book exam.	13	10.08%		
Total	129	100%		

Notably, 83 (64.34%) of the participants expressed a preference for open-book tests over CBEs. That is, 23 (17.83%) slightly preferred OBEs, 16 (12.4%) were neutral, one (0.78%) slightly preferred CBEs, and six (4.65%) preferred CBEs over OBEs, with a mean feeling score of 1.64 and a standard deviation of 1.05. This indicates a preference towards OBEs among the respondents (Table 3).

**Table 3:** Students' preferences: open-book vs. closed-book exams

How do you feel about open-book exams compared to traditional closed-book exams?	N	%	Mean	Std. deviation
Prefer open-book exams over closed-book exams.	83	64.34%	1.64	1.05
Slightly prefer open-book exams.	23	17.83%		
Neutral, no strong preference.	16	12.4%		
Slightly prefer closed-book exams.	1	0.78%		
Prefer closed-book exams over open-book exams.	6	4.65%		
Total	129	100%		

Concerning performance comparisons, 87 (67.44%) of the participants reported performing better on OBEs, 20 (15.5%) performed slightly better on OBEs, 14 (10.85%) stated similar performance on both OBEs and CBEs, five (3.88%) performed slightly worse on OBEs, and three (2.33%) performed worse on OBEs. The mean performance score is 1.58, and the standard deviation is 0.99 (Table 4).

**Table 4:** Participants' performance comparison: assessing open-book vs. closed-book exam methods

How did you perform compared to a traditional closed-book exam?	N	%	Mean	Std. deviation
I performed better on the open-book exam.	87	67.44%	1.58	0.99
I performed slightly better on the open-book exam.	20	15.5%		
My performance was similar on both open-book and closed-book exams.	14	10.85%		
I performed slightly worse on the open-book exam.	5	3.88%		
I performed worse on the open-book exam.	3	2.33%		
Total	129	100%		

These data illuminate students' sentiments regarding OBEs and their experiences with them. They also underscore a general preference for this assessment type, as students believe it reduces stress, requires less study time, accurately assesses their knowledge, and enhances critical thinking skills. This preference is particularly evident among medical students taking OBEs.

Assessment of self-directed learning using the OBE method

This section covered a wide range of topics related to self-directed study, particularly about OBEs. This section examined several essential topics, such as self-directed learning practices, strategies for exam preparation, opinions on assessment fairness, performance comparisons between OBEs and CBEs, and a thorough examination of the perceived benefits and drawbacks of OBEs for assessing self-directed learning.

Additionally, this section aimed to comprehend the pupils' viewpoints concerning the fairness of OBEs assessing self-directed learning. It also examined how students thought they performed in OBEs instead of CBEs, providing more information about their comfort level and aptitude for this type of evaluation.

Key findings

This research shed significant light on various aspects of self-directed learning and the impact of OBEs on students' approaches, attitudes, and performance. The results provide insightful information about how these tests affect learning strategies and perceptions of assessment fairness.

Exam preparation methods: 70 (54.26%) highlighted important points, 28 (21.71%) read the entire textbook, 28 (21.71%) created notes or summaries, two (1.55%) did all of the above, and one (0.78%) did nothing (Table 5).

**Table 5:** Strategies for preparing for open-book exams: a breakdown of students' approaches

How do you usually prepare for an open-book exam?	N	%
Highlight important points in the textbook	70	54.26%
Read the entire textbook before the exam	28	21.71%
Create notes or summaries of key concepts	28	21.71%
All of the above	2	1.55%
Nothing	1	0.78%
Total	129	100%

Fairness of OBEs for assessing self-directed learning: 85 (65.89%) believe it is a fair assessment, 32 (24.81%) are unsure, depending on the exams, eight (6.2%) think other methods are more suitable, and three (2.33%) have no opinion due to the lack of experience (Table 6).

**Table 6:** Students' perspectives on the fairness of open-book exams as indicators of self-directed learning

Do you feel that an open-book exam is a fair assessment of your self-directed learning abilities?	N	%
Yes, I believe an open-book exam is a fair assessment of my self-directed learning abilities	85	65.89%
I'm somewhat unsure, as it depends on the specific exam and subject.	32	24.81%
No, I think other assessment methods would be more suitable for measuring self-directed learning.	8	6.2%
I haven't taken enough open-book exams to form an opinion.	3	2.33%
Not applicable/not sure.	1	0.78%
Total	129	100%

Performance comparison: 87 (67.44%) performed better on OBEs, 20 (15.5%) performed slightly better, 14 (10.85%) had similar performance on both, five (3.88%) performed slightly worse, and three (2.33%) performed worse (Table 4).

Effect on self-directed learning: 85 (65.89%) strongly feel it encourages self-directed learning, 29 (22.48%) somewhat feel the encouragement, 11 (8.53%) neither encourages nor discourages, two (1.55%) somewhat discourages, and two (1.55%) strongly discourages (Table 7).

**Table 7:** Open-book exams' influence on self-directed learning: a detailed student perspective

Do you think that an open-book exam encourages or discourages self-directed learning?	N	%
Strongly encourages self-directed learning.	85	65.89%
Somewhat encourages self-directed learning.	29	22.48%
Neither encourages nor discourages self-directed learning.	11	8.53%
Somewhat discourages self-directed learning.	2	1.55%
Strongly discourages self-directed learning.	2	1.55%
Total	129	100%

## Discussion

Section 1

The study conducted an exhaustive and in-depth analysis of several aspects of students' experiences taking OBEs. This research attempted to provide a comprehensive picture of students' interactions with OBEs by delving into various aspects, such as stress levels, study techniques, comprehension of the subject, and the impact on self-directed learning.

Notably, 77 (59.69%) of the participants strongly agreed that OBEs cause less stress than typical CBEs. The reduction of performance pressure is one of the leading causes of this view [12]. By providing reference materials, OBEs foster a learning atmosphere where students feel less pressure to commit large amounts of knowledge to memory. Participants may feel more at ease due to the decreased need for memory since they may concentrate on comprehending and using concepts rather than memorizing facts by heart. Williams et al. further acknowledged that the transparency of open-book tests may help people feel less stressed [13]. Offering a safety net that lessens the worry of forgetting important information and reference materials may help participants feel more confident in their abilities to access and use them. A sense of empowerment may also be associated with less stress since OBEs are considered a more equitable evaluation. They let students show off their knowledge and problem-solving abilities without being unduly pressured to memorize answers [10]. Additionally, the general agreement on less stress during OBEs points to a benefit for students' general well-being. Given the growing emphasis on holistic development in educational techniques, it is critical to recognize and address the emotional components of evaluation. According to the findings, incorporating OBEs could be a calculated step toward developing a less demanding and more encouraging learning environment consistent with modern educational objectives.

The significant discovery that 71 (55.04%) of respondents strongly felt open-book tests accurately assessed their knowledge and understanding of the subject matter clarifies how effective this assessment technique is thought to be. This significant degree of agreement suggests that a sizable percentage of participants believe OBEs accurately indicate their comprehension and proficiency of the course material. According to Gharib et al., this impression is attributed to the idea that OBEs promote a more in-depth discussion of the material [14]. Participants can believe that searching through reference sources for pertinent information forces them to use critical thinking abilities, which shows a deeper understanding of the material. This trust in the efficacy of OBEs may also come from the assessment format's conformity with modern pedagogical ideas, which emphasize applying information and addressing real-world problems [15]. Moreover, there appears to be a positive association between open-book tests and perceived fairness, based on participants' faith in the assessment's capacity to measure knowledge and comprehension. OBEs offer a fair assessment that makes them more widely accepted as a legitimate and significant form of evaluation, underscoring their potential as a tactical element in gauging students' mastery of the material.

Additionally, 110 (85.27%) of participants said they prepared for OBEs in less time than CBEs. This may have a bearing on how easily accessible reference materials are and how influential people believe their preparation for OBEs to be [16]. The remarkable consensus among survey respondents underscores the generally acknowledged reduction in stress associated with OBEs and a general belief in the instructional use of this evaluation mode. Acknowledging less stress is noteworthy and suggests that the testing environment may change to one that is more encouraging and supportive. Furthermore, the widespread acceptance of the educational value of OBEs implies that students view them as valuable instruments for assessing their comprehension and application of the subject matter. The results suggest that using open-book tests in teaching procedures could be a calculated move to create a less stressful testing environment. By doing this, teachers can foster an atmosphere where students feel comfortable taking tests and their comprehension of the material is thoroughly assessed [17]. This dual advantage highlights OBEs' potential as a valuable and constructive form of assessment, supporting current initiatives to improve learning outcomes by reducing the stress related to conventional testing procedures.

Most participants (106, 82.17%) stated that they preferred open-book tests. The preference for open-book tests among participants can be attributed to the combined benefits of reduced anxiety and more effective study sessions. According to Theophilides et al., the appeal of these tests is that they reduce test-related anxiety since they allow people to refer to resources, which builds confidence [2]. The traditional closed-book method frequently causes tension and heightens the worry that important information may be forgotten when things become busy. On the other hand, OBEs encourage a more laid-back environment and let students concentrate on using their knowledge rather than memorizing facts by heart. The preference emphasizes the idea that there are more advantages to having access to reference materials than disadvantages. Participants understand that real-world situations frequently allow for resource consultation, demonstrating a pragmatic approach to evaluation. This reflects a change in educational philosophy that emphasizes assessing students' capacity to synthesize knowledge from various sources and their memorizing abilities. Exams with open books support the modern emphasis on problem-solving and critical thinking instead of fact memorization.

Another essential component of OBEs is the reduced preparation time. Study strategies can be more focused, with an emphasis on conceptual comprehension as opposed to memorization of specifics. This lessens the workload for students while reflecting work settings where making effective use of resources is crucial [12]. The inclination towards OBEs is a calculated decision by the participants, who see the value of realistic, stress-relieving evaluation techniques that align with real-world requirements.

The noteworthy discovery that 80 (62.02%) of participants strongly agree that OBEs foster critical thinking also reveals a fundamental component of the benefits participants perceive from this type of assessment. The participants' recognition of OBEs as facilitators of higher-order thinking skills is highlighted by this unanimity. Recognizing this cognitive advantage implies that participants think open-book tests are essential to developing advanced intellectual capacities, going beyond the traditional assessment objective of gauging knowledge retention. It is implied by the promotion of critical thinking that OBEs force students to interact more deeply with the subject matter [2]. Participants value the chance to analyze, synthesize, and apply information more sophisticatedly rather than depending only on memorization. This aligns with current educational goals, which highly value acquiring analytical abilities and the capacity for critical thought in various settings. For educational institutions and instructors, the apparent benefit of OBEs in fostering critical thinking is a crucial realization. It emphasizes that assessment techniques are essential for fostering students' analytical thinking and measuring their factual knowledge to align with broader educational objectives [18]. Due to this, these results might force a reevaluation of assessment methods, possibly leading to a move toward more OBEs to meet better the goals of encouraging students to use higher-order thinking abilities.

Overall, the results show that medical students often favor OBEs, which can be explained by their perceived advantages in lowering stress, requiring less time to study, efficiently evaluating knowledge, and promoting critical thinking. These important realizations highlight the benefits of OBEs and how they affect medical students' educational experiences.

Section 2

The results of this study provide important new information about how well OBEs work as an evaluation tool when used in conjunction with self-directed learning. The findings show that a sizable majority of students use a variety of ways to prepare for exams, with reading the complete textbook, underlining key ideas, and making notes or summaries being the most popular approaches. The variety of preparation techniques demonstrates how flexible students may be in self-directed learning settings since they can modify their strategies to fit their learning preferences.

Students' overwhelming agreement - 117 (90.7%) of them - that OBEs are an appropriate way to evaluate self-directed learning indicates a significant consensus. This resounding endorsement points to a consensus that OBEs provide a fair and impartial framework for assessing students' comprehension and application of the material. According to Dave et al., the substantial degree of agreement most likely stems from the belief that OBEs align with the fundamental ideas of self-directed learning [19]. Students appear to value how open-book evaluations support the fundamental principles of self-directed learning by emphasizing understanding, critical thinking, and applying knowledge practically [20]. Open-book tests require a better comprehension of the material than typical CBEs, which frequently rely on memorization. Students view these tests as opportunities to demonstrate their proficiency in navigating and using resources, promoting a more all-encompassing instruction method.

In the context of self-directed learning, the performance comparison between OBEs and CBEs offers strong support for the effectiveness of open-book evaluations. A significant proportion of students reporting either marginally better or better performance on OBEs supports the idea that these tests help students understand the content at a deeper level. This pattern supports the idea that open-book tests help students better comprehend the material by requiring them to do more than memorize facts [4].

Students can access various reference materials during the assessment process, instead of depending only on memorization. This method emphasizes the capacity to find, assess, and apply information successfully. It also conforms to the principles of self-directed learning and mirrors real-world problem-solving scenarios. Loi et al. argued that the positive performance results on OBEs support a paradigm change in assessment, acknowledging the importance of evaluating students' abilities to explore and apply their knowledge in real-world contexts in addition to their content knowledge [21]. Thus, in self-directed learning, open-book evaluations catalyze and encourage critical thinking, creativity, and a deeper comprehension of the material.

OBEs have a crucial impact on self-directed learning; most participants strongly agreed that they encourage self-directed learning. This positive correlation might be ascribed to the intrinsic features of OBEs, which require students to use their critical thinking, problem-solving, and thorough knowledge of the material [5]. OBEs require students to explore and synthesize material from multiple sources, encouraging a more comprehensive and integrated approach to learning than closed-book ones, which may promote rote memorization. OBEs align with the self-directed learning concept, as evidenced by the positive association between these assessments and the ideas of student autonomy and personal accountability for their own education. Brightwell et al. argued that, since OBEs prioritize application and synthesis of knowledge above memorization, they become an effective tool for evaluating understanding and developing the abilities necessary for self-directed learners in real-world situations [16].

For a more complex view of assessment preferences, it is essential to recognize the minority of participants who said they were neither encouraged nor deterred by open-book tests. The variation in responses highlights the range of unique learning preferences and styles among students. Even though most people believe that OBEs encourage independent study, instructors must acknowledge and value the diversity of viewpoints. These results highlight how crucial it is to consider different learning styles to guarantee inclusion and equity in assessment procedures. Johanns et al. argued that teachers should think about providing a range of assessment techniques to meet the diverse requirements of their students [6]. Different students engage with and display their mastery of the content differently, and a one-size-fits-all strategy may only work for some students. This recognition of individual variations makes teaching strategies more flexible and promotes an inclusive learning environment that meets the varied needs and preferences of the student body.

**Figure 1:**
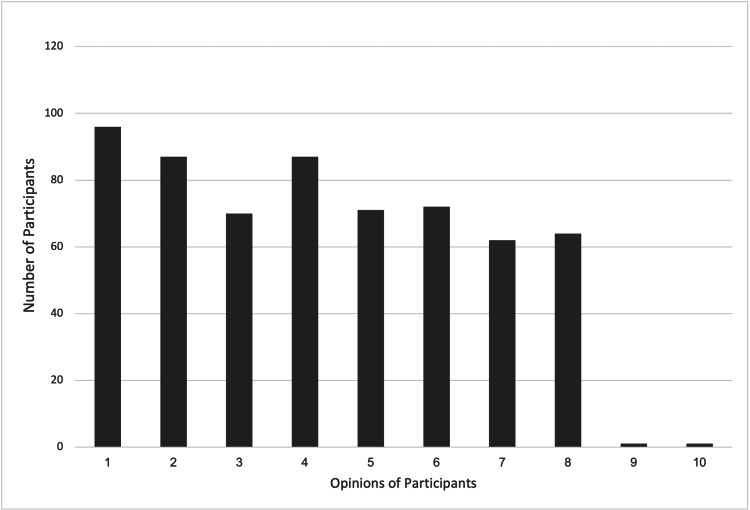
Participants' views on the disadvantages of open-book exams (OBEs) The numbers on the X-axis represent the following. (1) It may lead to over-reliance on reference materials rather than understanding. (2) It could make the exam too time-consuming for some students. (3) It may not effectively assess a student's ability to retain information. (4) It might not be suitable for subjects that require strict memorization. (5) It may create a sense of ambiguity about what is allowed during the exam. The Y-axis represents the number of participants.

**Figure 2:**
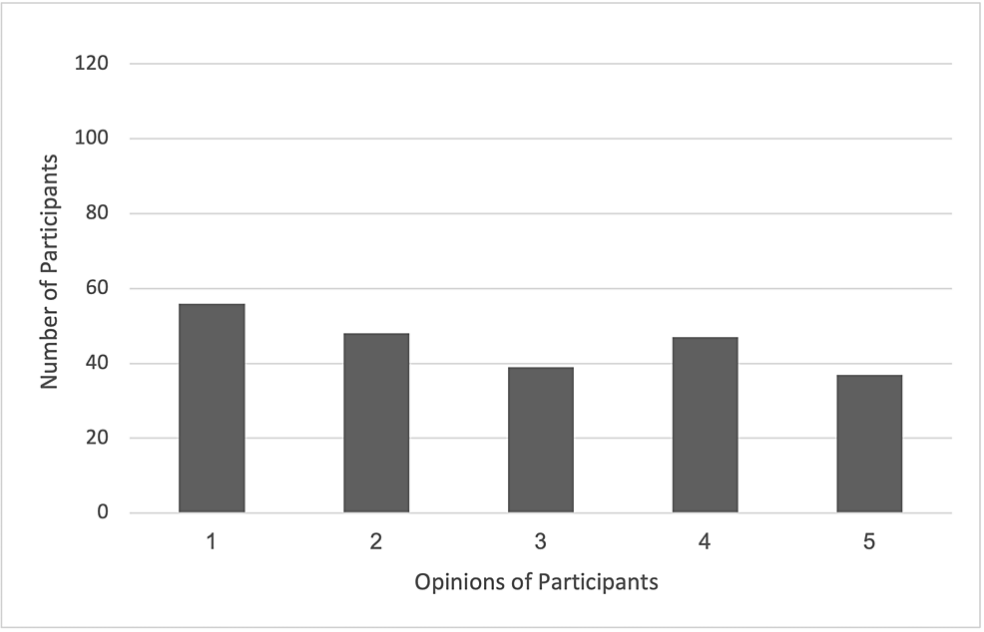
Participants' views on the advantages of open-book exams (OBEs) The numbers in the X-axis represent the following. (1) It promotes a deeper understanding of the subject matter. (2) It encourages students to develop critical thinking skills. (3) It simulates real-world problem-solving scenarios. (4) It reduces the pressure associated with memorization. (5) It allows students to access and apply reference materials, mimicking real-life situations. (6) It allows for greater flexibility and creativity in answering questions. (7) It encourages students to develop strong note-taking and organizational skills. (8) It promotes critical thinking and problem-solving abilities. (9, 10) Other. The Y-axis represents the number of participants.

Finally, participants admitted that using OBEs in the context of self-directed learning has certain drawbacks. Implementing this evaluation method requires considerable thought because of concerns about time consumption, suitability for subjects that require memorization, and the risk of overreliance on reference materials. These restrictions imply that OBEs might not be appropriate for all situations and could need to be carefully modified depending on the learning objectives and the subject matter (Figures 1, 2).

These results demonstrate, in general, the beneficial effects of OBEs on students' performance, learning tactics, and perceptions of these tests as encouraging self-directed learning practices. These revelations have essential ramifications for instructional tactics and academic assessment practices.

Nevertheless, this study is subject to some limitations. It relies on self-reported data, potentially introducing response bias. Additionally, the predominantly single-institution sample may compromise generalizability. Despite efforts to ensure participant anonymity and address ethical considerations, the findings may not fully encompass the varied perspectives within the medical student population.

Recommendations

OBEs provide benefits by allowing students to learn independently and explore materials to gain deeper comprehension. This method encourages critical thinking as students work through a variety of materials. However, the possibility for time management errors and the preference for external resources over internalized knowledge are disadvantages. In order to preserve fairness and avoid over-reliance, respondents emphasize the significance of having explicit guidelines on resource usage. To ensure that the exam accurately measures understanding, improvements should concentrate on developing questions that measure critical thinking abilities rather than memorization. Practice opportunities also facilitate students' effective navigation of open book formats, improving their capacity to apply knowledge in real-world contexts.

## Conclusions

This comprehensive study explores medical students' complex feelings and perspectives about OBEs, providing insight into how effective they are at encouraging self-directed learning. The study's conclusions are significant for medical education educators, organizations, and curriculum designers. The advantages of OBEs, such as their capacity to foster critical thinking and self-directed learning, highlight their potential as valuable instruments for evaluation. On the other hand, difficulties with time management and the potential for resource overuse point to the necessity of cautious execution and support systems. Future evaluation tactics will be shaped in part by the research's suggestions, which emphasize the need for explicit guidelines about resource usage and the inclusion of questions that encourage critical thinking. In the end, the study contributes substantially to the continuing discussion about improving medical education evaluation techniques to align with students' changing requirements and the changing environment of healthcare practices.

## Appendices

**Table 8:** Questionnaire

Questionnaire
Section 1: Medical students' experience with open-book exams:
Open-book exams are less stressful than traditional exams:
A. Strongly agree
B. Agree
C. Neutral
D. Disagree
E. Strongly disagree
Open-book exams accurately assess my knowledge and understanding of the material:
A. Strongly agree
B. Agree
C. Neutral
D. Disagree
E. Strongly disagree
It is easier to prepare for an open-book exam compared to a closed-book exam:
A. Strongly agree
B. Agree
C. Neutral
D. Disagree
E. Strongly disagree
How much time did you spend studying for the open-book exam compared to a traditional closed-book exam?
A. I spend more time studying for the open-book exam compared to a traditional closed-book exam.
B. I spend less time studying for the open-book exam compared to a traditional closed-book exam.
C. I spend the same time studying for the open-book exam compared to a traditional closed-book exam.
Open-book exams allow you to demonstrate your knowledge better than traditional exams:
A. Strongly agree
B. Agree
C. Neutral
D. Disagree
E. Strongly disagree
Open-book exams are a fair way to assess student knowledge:
A. Strongly agree
B. Agree
C. Neutral
D. Disagree
E. Strongly disagree
I feel like open-book exams are a fair way to assess student knowledge:
A. Strongly agree
B. Agree
C. Neutral
D. Disagree
E. Strongly disagree
How do you feel about open-book exams compared to traditional closed-book exams?
Prefer open-book exams over closed-book exams.
Slightly prefer open-book exams.
Neutral, no strong preference.
Slightly prefer closed-book exams.
Prefer closed-book exams over open-book exams.
The open-book format encourages you to think more critically about the material:
A. Strongly agree
B. Agree
C. Neutral
D. Disagree
E. Strongly disagree
How do you approach studying for an open-book exam? Do you focus on memorization or understanding the concepts?
A. Memorization concept
B. Understanding concept
C. I don’t know
D. Others (please specify)
Section 2: Assessment of self-directed learning using open-book exam method:
How do you usually prepare for an open-book exam?
A. Read the entire textbook before the exam
B. Highlight important points in the textbook
C. Create notes or summaries of key concepts
D. Other (please specify).
Do you feel that an open-book exam is a fair assessment of your self-directed learning abilities?
A. Yes, it allows me to demonstrate my understanding of the material in a practical way.
B. No, it doesn't accurately reflect my ability to recall information without external resources.
C. Unsure.
How did you perform compared to a traditional closed-book exam?
A. I performed better on the open-book exam.
B. I performed worse on the open-book exam.
C. There was no significant difference in my performance.
Do you think that an open-book exam encourages or discourages self-directed learning?
A. Encourages - it allows me to explore and learn more deeply about the subject matter.
B. Discourages - it makes me rely too much on external resources instead of retaining information from memory.
C. Unsure.
In your opinion, what are some advantages of using an open-book exam for assessing self-directed learning?
A. It allows for greater flexibility and creativity in answering questions.
B. It encourages students to develop strong note-taking and organizational skills.
C. It promotes critical thinking and problem-solving abilities.
D. Other (please specify).
What are some disadvantages of using an open-book exam for assessing self-directed learning?
A. Students may become too reliant on external resources instead of retaining information from memory.
B. It may be difficult to design questions that accurately assess understanding rather than just memorization.
C. It may be more time-consuming to grade open-book exams.
D. Other (please specify).
Do you have any suggestions for improving the use of open-book exams as a method of assessing self-directed learning?
A. Provide clear guidelines and expectations for how external resources can be used during the exam.
B. Design questions that require critical thinking and problem-solving rather than just memorization.
C. Provide opportunities for students to practice taking open-book exams before the actual assessment.
D. Other (please specify).
